# Sotos syndrome treated with traditional Chinese medicine and rehabilitation: Case report

**DOI:** 10.1097/MD.0000000000036169

**Published:** 2023-12-01

**Authors:** Si Chen, Pinfang Zou, Liyuan Ge, Xinran Cheng

**Affiliations:** a Department of Children Genetics and Endocrinology, Chengdu Women’s and Children’s Central Hospital, School of Medicine, University of Electronic Science and Technology of China, Chengdu, China; b Department of Children Rehabilitation, Chengdu Women’s and Children’s Central Hospital, School of Medicine, University of Electronic Science and Technology of China, Chengdu, China.

**Keywords:** microdeletion, rehabilitation, Sotos syndrome, traditional Chinese medicine

## Abstract

**Rationale:**

Sotos syndrome is an congenital overgrowth syndrome characterized by the primary features including overgrowth, distinctive facial features, learning disability, and accompanied with various second features. NSD1 deletion or mutation is a major pathogenic cause. Although there are some reports on treatment of this disease worldwide, less cases under treatment have been published in China.

**Patient concerns:**

A 1-year-old boy had macrocephaly, gigantism, excessive high body height, a particular face and delayed development, with a pathogenic gene of NSD1 (NM_022455.5:c.3536delA in exon 5).

**Diagnosis and interventions:**

The child was definitely diagnosed as Sotos syndrome and have 3 months’ combination treatment of traditional Chinese medicine and rehabilitation.

**Outcomes:**

The child made a great progress in global development.

**Lessons:**

This case firstly describes the traditional Chinese medicine and rehabilitation to treat Sotos syndrome in China. There is no radical cure, but our therapy could improve the prognosis and the life quality of the patient. Therefore, this case provides a reference to the clinical treatment of Sotos syndrome.

## 1. Introduction

Sotos syndrome is a rare genetic disease of autosomal dominant condition. In 1964, Sotos et al found 5 patients with characteristic facial appearances and overgrowth though the present authors suspect the case reported by Schlesinger in 1931 may have been firstly described.^[1]^ Sotos syndrome is a congenital overgrowth disorder with an incidence of approximately 1 in 14,000 live births.^[2]^ There is currently no exact prevalence in China. According to a review of the literature until July 2023, hundreds of cases have been reported. Although autosomal dominant pedigrees were reported, most of the cases are still sporadic.^[3]^

The pre- and postnatal overgrowth is Sotos early phenotype, and then characterized by a typical facial appearance, developmental delay, neurological disorder and advanced bone age so on.^[4]^ In the first year, the majority of the patient have a macrocephaly with over 97th percentile occipitofrontal circumference (OFC) and height, so that gigantism persisted into adulthood reported in a Dutch family.^[3]^ During the puberty, the overgrowth will slow down tending to normalize, and the height is normal in the most of adults.

However, It is still under known. Hence the article reports a case of Sotos syndrome in a 1-year-old child with normal birth weight, whose cognitive and motor functions were significantly improved under a combination of traditional Chinese medicine and rehabilitation training.

## 2. Ethic

This report obtain written informed consent from the parent of this patient. The institutional ethical committees approved this research.

## 3. Case report

A 1-year-old boy presented with macrocephaly had been paid attention since 3 months of age, following by delay gross motor. Child being 41 weeks gestational age was born by eutocia turning to cesarean section with a 3.85 kg birth weight and a birth body length of 54 cm, but head circumference were not measured, and then hospitalized because of neonatal jaundice and hypoglycemia. At his 4 months of age, the first tooth erupted. Weight, body length and OFC were examined every 1 or 2 months and plotted on World Health Organization growth charts and body length were found over the normal since his birth. Until 2nd and 3rd month of life, his weight and head circumference start to be growing quickly above the 97th percentile respectively (Table 1). He has a high anterior hairline, frontotemporal sparseness of hair, broad forehead, a long face, pointed mandible and long extremities. His mother had a similar dysmorphic characteristics too. During half a year old, he could not grip actively, sit down alone and turn over, so he was admitted to the rehabilitation department of our hospital. The reexamination of echocardiography was decreased to 1.6 mm patent foramen ovale from 3.20 mm at the age of 3 months. There were high blood lactic acid 4.83 mmol/L and FT3 4.91 pg/mL with a low hypersensitive troponin 0.009 ng/mL, but blood routine, routine urine test, liver function, lipid, homocysteine, ammonia, folate 22.07 ng/mL and coagulation function are normal. Electroencephalogram, visual evoked potential, auditory evoked potential, and pelvis X ray were normal. MRI brain showed ventriculomegaly, thin corpus callosum, widening of bilateral frontotemporal internal and external space. DTI brain appeared that fibers of the bilateral corpus callosum were partially interrupted and did not reach the cortex; left corticospinal tract was slightly sparse, right corticospinal tract ran to the medulla oblong and was far sparse, right cingulate tract was sparse compared with the contralateral tract, the anterior part of the left arcuate tract was locally interrupted; and the fibers of the right inferior fronto-occipital tract did not reach the cortex. Gesell Developmental Schedules reflected that delayed adaptive behavior, gross motor, fine motor, language, personal-social behavior scored 62, 49, 53, 45, and 61 respectively. In the light of prominent facial features and developmental delay, we advised the proband for gene detection including whole exome sequencing, copy number variations, mitochondrial mutation and fragile X chromosome. The final result was a deletion variant of NSD1 in heterozygous status for autosomal dominant disease. The variant was NM_022455.5:c.3536del in exon 5. Namely, one-base deletion (3536delA) in exon 5 results in the change of amino acid at position 1179 from glutamic acid to glycine, and then produces a truncation mutation (1217 aa), thus leads to the termination of protein synthesis. According to ACMG, it was pathogenic variant. Mother was confirmed to be heterozygous and evaluation was advised. Hence the child was definitely diagnosed as Sotos Syndrome. The proband was treated with physio therapy, occupational therapy and speech therapy, combined with scalp-acupuncture, needle-embedding therapy and auricular acupuncture. After 3 month combination therapies, Gesell Developmental Schedules was improved on higher scores 57, 62, 59, 51, and 64 respectively above. Specifically, the child made a great progress in gross motor and language characterized by active position change, sit down and turn over. To date the child has been receiving rehabilitation.

**Table 1:** Weight, length and OFC measured in the different age stage.

Age	OFC (cm)	Body height (cm)	Body weight (kg)
At birth	–	54.00 (>97th percentile)	3.85
1 mo	38.80	59.50 (>97th percentile)	5.45
2 mo	41.20	64.40 (>97th percentile)	7.10 (>97th percentile)
3 mo	43.80 (>97th percentile)	68.30 (>97th percentile)	8.24 (>97th percentile)
4 mo	45.00 (>97th percentile)	69.00 (>97th percentile)	9.23 (>97th percentile)
6 mo	48.00 (>97th percentile)	75.00 (>97th percentile)	11.10 (>97th percentile)
11 mo	50.30 (>97th percentile)	80.30 (>97th percentile)	11.90 (>97th percentile)
12 mo	50.00 (>97th percentile)	83.50 (>97th percentile)	12.00 (>97th percentile)

mo = month, OFC = occipitofrontal circumference.

## 4. Discussion

Sotos syndrome (OMIM 117550) is also known as cerebral gigantism. Isolation and characterization of human homologue NSD1 (Nuclear receptor-binding SET domain containing protein 1) revealed that it contains more than 23 exons, has an 8088 bp open reading frame, encodes 2696 amino acids and is expressed in the fetal/adult brain, kidney, skeletal muscle, spleen, and the thymus, and faintly in the lung.^[5]^ The majority of Sotos syndrome is ascribed to mutations and deletions of NSD1, encoding the histone methytransferase to regulate chromatin, which has led to a reevaluation of definition and manifestations related to this disease.^[2]^ Haploinsufficiency of NSD1 is reported to the major cause of Sotos syndrome, and NSD1 acts as a co-repressor (as opposed to a co-activator) of genes that promote growth.^[6]^ Our case is also a microdeletion caused haploinsufficiency of NSD1, which induces overgrowth in Sotos syndrome.

Most facial appearances are prominent, presenting a high anterior hairline, frontotemporal sparseness of hair, broad forehead, inverted triangular face, pointed mandible and long extremities, down slanting palpebral fissures, apparent hypertelorism, red zygomatic, macrotia and a high palate.^[7,8]^ The chin is the most distinctive facial feature in adulthood.^[2]^ Overgrowth is coming to the forth in infancy. In detailed, the patient has an excessive growth in the physical examination parameters including body height and OFC. Concomitant with gigantism and macrocephaly, the height and OFC are over the 97th percentile. However, most of these patients are excessively tall only from the birth to childhood, but the combination of advanced bone age and early onset of menarche leads to a final height within the normal range for most population after puberty.^[9]^ Consequently, height tends to normalize in adulthood, but macrocephaly continues to exist at any age stage. The majority of patients have learning difficulties. Most individuals have mild or moderate intellectual impairment but the impaired degree is very broad, ranging from occasional individuals with normal development to these with serious learning disability requiring life-long care.^[10]^ Other features include neonatal jaundice, feeding difficulties, scolisis, hearing impairment, strabismus, nystagmus, heart defects, seizures, hypotonia, adaptive problems in social functioning, some present autistic features, genitourinary abnormalities, neoplastic and other symptoms.^[3,11]^

At present, there are still unknown guidelines or consensus on diagnostic criteria for Sotos syndrome. As numerous cases of Sotos syndrome are reported, the accepted diagnostic criteria is becoming more detailed. At the earliest, Cole et al suggested a major diagnostic criteria for Sotos syndrome based on an analysis of 79 children: particular facial gestalt, overgrowth pattern, advanced bone age, and developmental delay.^[1]^ Tatton-Brown K et al added a new interpretation to the diagnosis of the disease, using molecular genetic testing, including single-gene testing or use of a multigene panel on the basis of the Cole’ criteria.^[2,12]^ In the latest study, Tatton-Brown K also elaborated that Sotos syndrome had the cardinal features characterized by a distinctive facial appearance, learning disability and overgrowth, and major features including behavioral findings (most notably autistic spectrum disorder), advanced bone age, cardiac anomalies, cranial MRI/CT abnormalities, joint hyperlaxity with or without pesplanus, maternal preeclampsia, neonatal complications, renal anomalies, scoliosis, and seizures.^[13]^ As noted, special facial appearance, overgrowth and mental retardation are critical to diagnosis, but the features of partial patients are not typical. Therefore, unusual manifestations need more reported, paid attention and distinguished from other overgrowth diseases by genetic testing, such as Weaver syndrome, Beckwith-Wiedemann syndrome, Simpson-Golabi-Behmel syndrome, Klinefelter syndrome, Marfan syndrome, Bannayan-Riley-Ruvalcaba syndrome, Kalmann syndrome and Perlman syndrome.

After diagnosis, management needs multidisciplinary participation for symptomatic therapies. In the first month after birth, there are supplemental glucose treating hypoglycemia, phototherapy deletion of jaundice, and other therapies to deal with feeding problems and gastroesophageal reflux so on. During the following year, periodic physical examination is necessary in the department of Pediatric Outpatient Preventative Care to find overgrowth and delayed development. Meanwhile, some clinical signs and complications may occur such as acromegalia, advanced bong age, scoliosis and seizures. The timely symptomatic treatment and adequate rehabilitation act as a important part to improve the global development of the patients and poor prognosis. The psychological and educational program with speech therapy and motor stimulation are recommended.^[14]^ The high body height only needs to measure in period because the final will get to normal height in adulthood. Nonetheless, it is crucial to allow detection and management of tumorigenesis during the patient’s lifetime.

## 5. Conclusion

Sotos syndrome is a non-progressive neurologic disorder caused by NSD1 deletions or mutations. It is a clinical diagnosis based on major criteria described above. In the study, the child had very common clinical manifestations, that is, large skull, a long thin face, premature eruption of dentition, cardiac defect, overgrowth and developmental delay. In pediatric follow-up visit, the facia gestalt and overgrowth is not difficult to find. Thereby, early diagnosis, genetic counsel, traditional Chinese medicine therapy and rehabilitation play a fundamental role. This case is a better example and also provide a referred value in degree for clinical management.

## Acknowledgments

The authors greatly appreciated the patient and his parents participated in the study. The authors are indebted to Beijing Mygenostics Co., Ltd. for Sanger sequencing and technical assistance.

## Author contributions

**Conceptualization:** Si Chen.

**Data curation:** Liyuan Ge.

**Formal analysis:** Liyuan Ge.

**Funding acquisition:** Si Chen.

**Methodology:** Pinfang Zou.

**Project administration:** Pinfang Zou.

**Supervision:** Xinran Cheng.

**Visualization:** Xinran Cheng.

**Writing – original draft:** Si Chen.

**Writing – review & editing:** Si Chen, Pinfang Zou, Liyuan Ge, Xinran Cheng.
